# Increased Right Posterior STS Recruitment Without Enhanced Directional-Tuning During Tactile Motion Processing in Early Deaf Individuals

**DOI:** 10.3389/fnins.2020.00864

**Published:** 2020-08-25

**Authors:** Alexandra N. Scurry, Elizabeth Huber, Courtney Matera, Fang Jiang

**Affiliations:** ^1^Department of Psychology, University of Nevada, Reno, Reno, NV, United States; ^2^Department of Speech and Hearing Sciences, Institute for Learning & Brain Sciences, University of Washington, Seattle, WA, United States

**Keywords:** cross-modal plasticity, early deafness, superior temporal sulcus, auditory cortex, tactile motion

## Abstract

Upon early sensory deprivation, the remaining modalities often exhibit cross-modal reorganization, such as primary auditory cortex (PAC) recruitment for visual motion processing in early deafness (ED). Previous studies of compensatory plasticity in ED individuals have given less attention to tactile motion processing. In the current study, we aimed to examine the effects of early auditory deprivation on tactile motion processing. We simulated four directions of tactile motion on each participant’s right index finger and characterized their tactile motion responses and directional-tuning profiles using population receptive field analysis. Similar tactile motion responses were found within primary (SI) and secondary (SII) somatosensory cortices between ED and hearing control groups, whereas ED individuals showed a reduced proportion of voxels with directionally tuned responses in SI contralateral to stimulation. There were also significant but minimal responses to tactile motion within PAC for both groups. While early deaf individuals show significantly larger recruitment of right posterior superior temporal sulcus (pSTS) region upon tactile motion stimulation, there was no evidence of enhanced directional tuning. Greater recruitment of right pSTS region is consistent with prior studies reporting reorganization of multimodal areas due to sensory deprivation. The absence of increased directional tuning within the right pSTS region may suggest a more distributed population of neurons dedicated to processing tactile spatial information as a consequence of early auditory deprivation.

## Introduction

Individuals affected by early sensory deprivation often display enhanced perceptual sensitivities for the remaining modalities. For instance, visual motion detection (Shiell et al., 2014) and visual motion direction discrimination (Hauthal et al., 2013) appear to be superior in early deaf (ED) participants compared to normal hearing (NH). These behavioral changes are typically accompanied by cross-modal reorganization, where brain areas deprived of default sensory input respond to input from the remaining modalities. Prior studies with ED adults report activation of primary auditory cortex (PAC) to peripheral visual stimuli (Karns et al., 2012), during presentation of visual motion (Finney et al., 2001; Fine et al., 2005) and during a visual rhythm matching task (Bola et al., 2017). Other studies report similar cross-modal reorganization of auditory cortex in ED for processing vibrotactile stimuli (Levänen and Hamdorf, 2001; Auer et al., 2007) and during touches to the face (Karns et al., 2012).

Often, cross-modal plasticity follows the concept of functional constancy – deprived cortical areas retain function but shift the type of sensory input (Bavelier and Neville, 2002; Amedi et al., 2007; Saenz et al., 2008; Jiang et al., 2014; Renier et al., 2014). For instance, when presented with both fixed-frequency and speech-derived vibrotactile stimuli, more widespread activity within auditory region was observed in ED participants (Auer et al., 2007). In addition, distinct patterns of activity were observed for a deviant vibrotactile frequency compared to a standard vibrotactile frequency in supratemporal auditory cortex of an ED case study participant (Levänen et al., 1998). Auditory signals normally convey similar temporal and frequency information as vibrotactile stimuli, as evidenced by robust interaction between these two modalities when their frequencies overlap (Crommett et al., 2017; Pérez-Bellido et al., 2017). Indeed, specificity toward vibrotactile frequency demonstrated in the above findings provides evidence of maintained auditory cortex function in ED adults for haptic rather than auditory sensory input.

Along with cross-modal recruitment of primary sensory areas, multimodal regions, such as the posterior superior temporal sulcus (pSTS), are additional targets for compensatory plasticity upon early auditory deprivation because multimodal areas exhibit increased numbers of neurons responsive to intact modalities when one modality is deprived (Rauschecker and Korte, 1993; Meredith et al., 2011). The pSTS is an association area within the superior temporal cortex that normally displays responses to multiple modalities including auditory, visual, and tactile (Beauchamp et al., 2008; Jiang et al., 2015) and is involved in the perception of biological motion (for review see Decety and Grèzes, 1999; Grossman et al., 2000; Grossman and Blake, 2002). Sadato et al. (2004) reported that early deaf (< 2 years), late deaf (> 5 years), and NH individuals all showed activation of the pSTS during visual processing of sign language. However, only ED adults demonstrated increased activity of the middle STS (Sadato et al., 2004), a region normally responsive to voices and audiovisual speech during speech processing (Venezia et al., 2017). Such findings suggest that, in response to early deafness, inherently auditory or multimodal STS areas demonstrate cross-modal reorganization and enhanced activation for the visual modality to retain STS function, in this case linguistic processes. In addition to decoding auditory and visual features of language, the pSTS region is also involved in audiovisual temporal processing (Zhang et al., 2010; Noesselt et al., 2012) and visual motion processing (Beauchamp et al., 2002; Grossman and Blake, 2002; Nelissen et al., 2006). Therefore, it is not surprising that bilateral superior and middle temporal gyri activation was evident in ED adults during a visual rhythm discrimination task (Bola et al., 2017), and STS activation patterns were dependent on the frequency of a vibrotactile stimulus in an ED case study (Levänen et al., 1998).

While these various findings describe how an auditory-deprived brain functionally adapts its organization for visual motion processing, visual temporal processing (i.e., rhythm), and tactile temporal processing (i.e., frequency), changes to cortical processing of tactile motion as a result of early deafness remain untested. The higher-order processing of visual motion cues by the pSTS region, such as preferential activation for articulated vs. unarticulated human motion (Beauchamp et al., 2002), biological vs. scrambled motion (Grossman and Blake, 2002), and dynamic vs. static faces (Pitcher et al., 2011), establishes the pSTS as a likely candidate for cross-modal recruitment during motion processing tasks. Further, STS is responsive to auditory (Lewis, 2000), tactile (Jiang et al., 2015), and visual non-biological motion (Nelissen et al., 2006), allowing for a more salient and representative percept of the target of interest, a useful characteristic as the STS is heavily involved in processing social cues and interactions (Beauchamp, 2015; Deen et al., 2015; Venezia et al., 2017). Therefore, this study was particularly interested in characterizing the pSTS response to tactile motion upon early auditory deprivation.

Besides higher-order multisensory areas, we also wanted to examine any functional cross-modal reorganization of intact primary sensory areas in ED adults as these regions are crucial for processing tactile motion. Haptic motion discrimination and decoding the manner in which a tactile object moves across the skin is a crucial piece of information dictating our perception and understanding of the identity, function, and route of that object. Decoding of tactile motion direction is initiated by stimulation of rapidly adapting and slowly adapting type I afferents, which activate directionally tuned neurons localized within the subregions of macaque SI, specifically areas 3b, 1, and 2 (Pei et al., 2010; for review see Pei and Bensmaia, 2014), similar to the direction sensitivities of visual neurons located within primary visual cortex and MT + (Albright, 1984). Area 1 of SI plays a primary role in motion decoding as a large proportion of area 1 neurons demonstrate strong, coherence-dependent directional tuning, regardless of the type of tactile stimulus (Pei et al., 2010). Comparable to models describing the mechanism of global visual motion perception in MT + (Amano et al., 2009, 2012), the convergence of tactile inputs to area 1 results in a global percept of tactile motion driven by the vector average of the two-dimensional contours comprising the plane of motion and the terminators, all weighted by their respective saliencies and speeds (Pei et al., 2011; for review see Pei and Bensmaia, 2014). The importance of SI in decoding tactile motion was further shown when transcranial magnetic stimulation applied to SI of NH adults resulted in a significant reduction in the ability to discriminate direction of tactile motion (Amemiya et al., 2017). In addition, the feed-forward inputs from SI to SII also contribute to the global percept of haptic features, including motion (Hsiao, 2008). Indeed, in NH individuals, both SI and SII reveal differential responses dependent on the direction of tactile motion stimulation (Wacker et al., 2011). As prior studies suggest reduced specificity for processing intact sensory inputs due to intramodal plasticity and computational efficiency (Gougoux et al., 2009; Stevens and Weaver, 2009; Jiang et al., 2014), it is likely that similar functional reorganization in ED SI/SII underlies processing tactile motion.

In addition to reorganization, cortical regions lacking input from their typical modality (i.e., PAC and auditory input) also undergo changes to tuning properties of their neuronal populations. In the anterior auditory field of ED cats, there was a shift in modality-specific neurons, as well as an increase in the visual and somatosensory neuronal receptive fields compared to NH cats, reflecting wider tuning of these neuronal populations (Meredith and Lomber, 2011), presumably allowing for greater compensation for the lost modality through a wider range of neural excitation. A similar finding in congenitally deaf cats revealed broader tuning of interaural time difference (ITD)–sensitive inferior colliculus neurons providing a probable explanation for the poor ITD discrimination common in cochlear implant users (Hancock et al., 2012; Laback et al., 2015). However, while ED adults exhibited a fivefold increase in multimodal pSTS activation compared to NH adults for directional visual motion, there was no evidence for direction specificity in active voxels (Retter et al., 2019), indicating that neuronal populations within the pSTS region may not demonstrate strong directional sensitivity for motion, or such profiles could not be elucidated with the frequency tagging approach used in that study.

As tuning properties of neuronal populations provide insight into the sensitivity and functional role of their respective cortical areas, an additional goal of the current study was to characterize the directional sensitivities in somatosensory regions and other areas that may exhibit reorganization for tactile processing in ED adults, such as PAC and pSTS region. In terms of tactile motion processing, direction discrimination is mediated by the directional sensitivity of neuronal populations within tactile processing areas (Hsiao, 2008; Pei et al., 2010, 2011; Hsiao and Gomez-Ramirez, 2011). Indeed, directional tuning of neurons within macaque SI displayed increased sensitivity to direction with increased motion coherence, a finding that closely resembled human behavioral performance on a tactile motion discrimination task (Pei et al., 2010). We used a modified population receptive field (pRF) analysis originally developed for retinotopic mapping (Dumoulin and Wandell, 2008) and later adopted for tonotopic mapping in PAC of NH individuals (Thomas et al., 2015) and in human middle temporal complex (hMT^+^) of early blind (EB) participants (Huber et al., 2019a, b). pRF estimation allowed for the characterization of tactile direction tuning profiles (directional selectivity and tuning bandwidth) of neuronal populations in areas of interest, specifically somatosensory cortices, PAC, and pSTS.

While we expected increased activation of pSTS for tactile motion in deaf due to the multimodal inputs inherent to this region and the loss of auditory input, we did not expect enhanced directional tuning in the pSTS of ED as Retter et al. (2019) previously reported absence of directional specificity for visual motion by ED despite increased pSTS activation. Indeed, we found enhanced pSTS activation by ED without an increase in the proportion of or changes in the bandwidth of directionally tuned voxels. In addition, we did not see greater activation of PAC by ED in line with our hypothesis based on the functional-constancy theory of cross-modal reorganization. Finally, we hypothesized similar activations of somatosensory areas in both ED and NH adults as this region’s primary sensory input is unaffected by early deafness while the tuning bandwidths of neuronal populations in SI and SII may be broadened in ED adults allowing for compensatory profiles of neural excitation. We did not find any differences in SI or SII activation between the two groups, whereas in the ED adults, we found reduced proportions of directionally tuned voxels in SI only.

## Materials and Methods

### Participants

Seven ED with bilateral severe to profound hearing loss (ages 31–55 years; two males; cause and age at onset of deafness are reported in Table 1) and 7 age- and gender-matched NH controls (ages 28–54 years) participated in this study. There was no statistical difference in age between the two groups (*t*_12_ = 1.04, *p* = 0.32). Participants were screened for any history of neurological or psychiatric disorders, history of brain injury, antipsychotic medications, and cognitive decline. Participants provided signed informed consent before any experimentation and were financially compensated for their time. Protocols were reviewed and approved by the institutional review board at the University of Nevada, Reno in accordance with the guidelines of the Declaration of Helsinki for research involving human subjects.

**TABLE 1:** Demographic information of early deaf participants.

Participant	Age (years)	Handedness	Clinical description	Age at deafness onset (months)	Auditory deprivation (left; right) (dB)
ED1	30–35	R	Fever	15	Total; 85
ED2	45–50	R	Maternal gestational measles	Birth	100; 90
ED3	30–35	R	Cytomegalovirus	12	Total; profound
ED4	40–45	R	Unknown	12	95; 95
ED5	30–35	R	Hereditary	Birth	80; 70
ED6	50–55	R	Unknown	Birth	85; 90–100
ED7	40–45	R	Spinal meningitis	9	Profound; profound

### Visual Motion Localizer

A visual motion localizer was used to identify pSTS region in all participants over a tactile motion localizer to avoid spurious results (Kriegeskorte et al., 2009; Jiang et al., 2015). Motion localizer scans consisted of blocks of moving and static dots as well as a fixation condition that did not contain any dots. Dots were presented within a circular aperture (radius 8°) with a central fixation cross surrounded by a gap (radius 1.5°) in the dot field. Visual stimuli were generated using MATLAB and PsychToolbox (Brainard, 1997; Pelli, 1997). Visual stimuli were back-projected onto a display located behind the magnet and viewed through a mirror attached to the MR head coil. All dots were white presented on a black background. Each dot subtended 0.3° (dot density 1 per degree). To prevent tracking of individual dots, the dots had a limited lifetime of 200 ms. In the moving condition, all dots moved coherently in one of eight directions (spaced evenly between 0° and 360°) with a speed of 8° per second. The direction of motion changed once per second, and the same direction never appeared in subsequent trials. In static conditions, the dots were presented without any motion, and the position of the dots was reset once per second. In fixation conditions, only the fixation cross was presented without any dots. Participants were asked to fixate throughout the scan without performing a task. Each block lasted 10 s during which one of the three visual stimulation conditions (motion, static, or fixation) was presented. Two motion localizer scans were obtained from every participant. Each scan lasted ∼5 min and included 30 10-s blocks.

### Tactile Stimulus Design and Procedure

**FIGURE 1:**
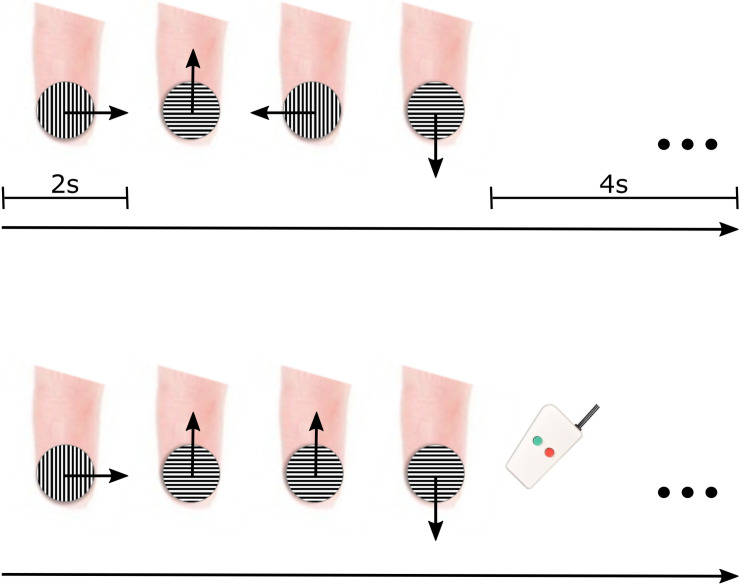
fMRI paradigm for delivering tactile motion. Each 12-s block consisted of all four directions of tactile motion (each direction was simulated for 2 s) followed by a 4-s baseline period (top panel). To ensure participant’s attention, two of the blocks had a direction immediately repeated, and participants were instructed to press a button on the response box when they noticed the repeat (bottom panel).

During tactile motion scans, motion was simulated in four main directions (rightward, leftward, upward, and downward) using a small grating surface held within a plastic tube (JVP dome) (Stoelting Co., Wood Dale, IL, United States) consisting of equidistant bar and groove widths equal to 0.35 mm. The JVP dome was manually placed on the center of the participant’s right index finger pad by an experimenter, and tactile motion was simulated by the experimenter sweeping the dome across the finger pad in the appropriate direction for a total of 2 s (1 sweep/1 s) (Figure 1). As displayed in Figure 1, the orientation of the dome was continually adjusted by the experimenter dependent on the direction of motion so that the orientation of the grooves embedded in the dome was perpendicular to the direction of motion. At the groove distance of 0.35 mm, participants were unaware of the dome’s orientation (Wong et al., 2011).

Each block of tactile motion contained all four directions. The order of directions was pseudorandomized to include all possible order combinations of tactile motion directions. Each block consisted of 8 s of tactile motion (2 s for each of the four directions of motion) followed by a 4-s baseline rest period (Figure 1, top panel). To maintain participant’s attention throughout each scan, they were asked to complete a 1-back task. In two of the blocks (7.69%), rather than presenting all four directions, one direction of tactile motion was randomly selected to be repeated (Figure 1, bottom panel). Upon perception of this direction repeat, participants were instructed to press a response button with their left hand. Each participant participated in four experimental scans. Each scan lasted ∼5 min and included 26 12-s blocks (including blocks containing the 1-back task). Participants wore an eye mask throughout tactile motion scans to prevent any visual input of the experimenter’s movements.

### fMRI Data Acquisition

Scanning was performed at the Neuroimaging Facility of Renown Health Hospital in Reno, NV on a 3T Philips Ingenia scanner using a 32-channel digital SENSE head coil (Philips Medical Systems, Best, Netherlands). Three-dimensional (3D) anatomical images were acquired at 1 × 1 × 1 mm resolution using a T1-weighted MPRAGE (magnetization-prepared rapid gradient echo) sequence. Functional images were obtained using a standard echo planar imaging sequence (EPI) with 2.75 × 2.75 × 3-mm voxels. A continuous block design was used (TR = 2 s, TE = 25 ms) for both visual motion localizer and tactile motion scans.

### Functional Magnetic Resonance Imaging Data Preprocessing

Data were analyzed using Brain Voyager QX (version 2.8; Brain Innovation, Maastricht, the Netherlands) and MATLAB (Mathworks, Natick, MA, United States). Initially, functional data underwent preprocessing steps that included three-dimensional motion correction (trilinear/sinc interpolation), high-pass filtering including linear trend removal [general linear model (GLM) approach with a design matrix containing a Fourier basis set (sines and cosines for two cycles)] and slice scan time correction (cubic spline). For each participant, preprocessed functional data were coregistered to their corresponding anatomical data. The initial alignment was based on header information from functional and anatomical sessions, and fine-tuning alignment was gradient based. Anatomical and functional data were then transformed into Talairach space (Talairach and Tournoux, 1988).

### Functionally Defined pSTS Region

**FIGURE 2:**
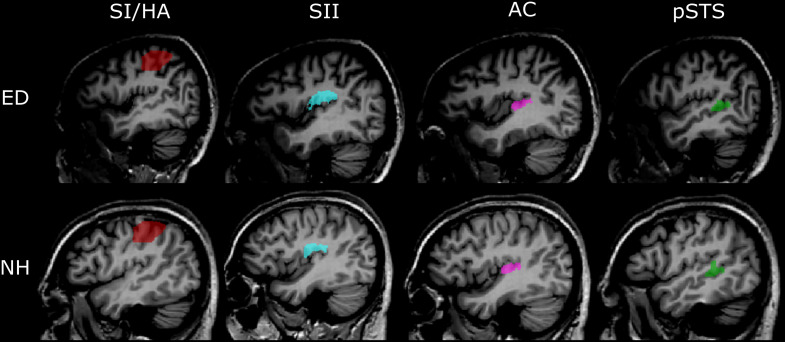
Representative anatomically and functionally defined ROIs. Sagittal views of the four ROIs in the left hemisphere defined using either the functional (pSTS) or anatomical (SI/HA, SII, PAC) criteria are shown in the Talairach volume space of a representative ED (top panel) and NH participant (bottom panel). Red, SI/HA; light blue, SII; pink, PAC; green, functionally defined pSTS.

To examine responses in the pSTS region, we functionally defined voxels that showed significant activation, based on a false discovery rate of 0.05 at the cluster level (qFDR < 0.05) averaged across all ED and NH participants to visual motion vs. static condition. However, pSTS recruitment for visual motion is primarily evident in ED, not NH participants; thus, this region of interest (ROI) did contain some bias for our ED group. Generous ROIs were created that encompassed superior STG, middle STG, and middle and posterior STS. The group-level pSTS region ROI was applied to individual volume space, and voxels were identified and removed if they encompassed part of the temporoparietal junction (TPJ), lateral fissure, or parietal operculum, resulting in individual pSTS region ROIs. Representative pSTS region ROIs from an ED and an NH participant are shown in Figure 2.

### Anatomically Defined ROIs

ROIs were created for primary somatosensory cortex/hand area (SI/HA), secondary somatosensory cortex (SII), and PAC using the Julich probabilistic atlas. We created maximum probability maps (Eickhoff et al., 2006) containing voxels from all subregions for SI, SII, and PAC (Figure 2). This procedure ensured no overlap between somatosensory regions or between SII and PAC as voxels could be assigned to only one ROI. Thus, voxels permitted to SII were prevented from also being assigned to PAC and vice versa. ROIs were then transformed to Talairach space and applied to each participant’s brain volume. Upon Talairach transformation, however, there was a small overlap between group-level SI and SII ROIs (30 and 31 functional voxels in the right and left hemispheres, respectively), as well as group-level PAC and SII ROIs (42 and 51 functional voxels in the right and left hemispheres, respectively). To ensure separate, non-overlapping SI/SII ROIS and SII/PAC ROIs within the Talairach space, overlapping voxels were removed. To limit the SI area for voxels encoding hand-specific information, the SI region was constrained along the *z* axis (coordinates between 37 and 63; Kitada et al., 2019). Central coordinates of the SI/HA ROIs are shown in Table 2.

**TABLE 2:** Talairach coordinates and total voxel number (in functional resolution) for group-defined ROIs.

ROI name	Hemisphere	*x*	*y*	*z*	No. of Voxels
SI/HA	R	32	−40	50	1066
	L	−35	−39f	49	1033
SII	R	49	−19	20	474.8 (43.83)
	L	−48	−18	18	562.3 (26.12)
PAC	R	45	−20	9	212.2 (14.82)
	L	−42	−22	9	192.0 (25.32)
pSTS region (functional)	R	51	−37	11	130.2 (11.81)
	L	−49	−37	6	90.4 (11.49)
pSTS region (anatomical)	R	52	−36	7	259.5 (23.64)
	L	−54	−37	6	246.2 (19.55)

*Numbers shown in parenthesis in the no. of voxels column are the SD. The right and left SI/HA ROIs were the same for all participants.*

In addition, structural volumes were used to manually identify and remove voxels from each individual’s SII ROI that were located on Heschl’s gyrus, along the planum temporale (PT), superior temporal gyrus (STG), or TPJ. Similarly, voxels from individual PAC ROIs were manually identified and removed if they extended beyond Heschl’s gyrus or superior temporal gyrus, or if they resided along the parietal operculum. These extra steps ensured that any overlap between SII and auditory cortex did not confound our analyses. Group averaged central coordinates and total number of voxels for SII and PAC ROIs are shown in Table 2. Representative SI/HA, SII, and PAC ROIs for an ED and NH participant are displayed in Figure 2.

**FIGURE 3:**
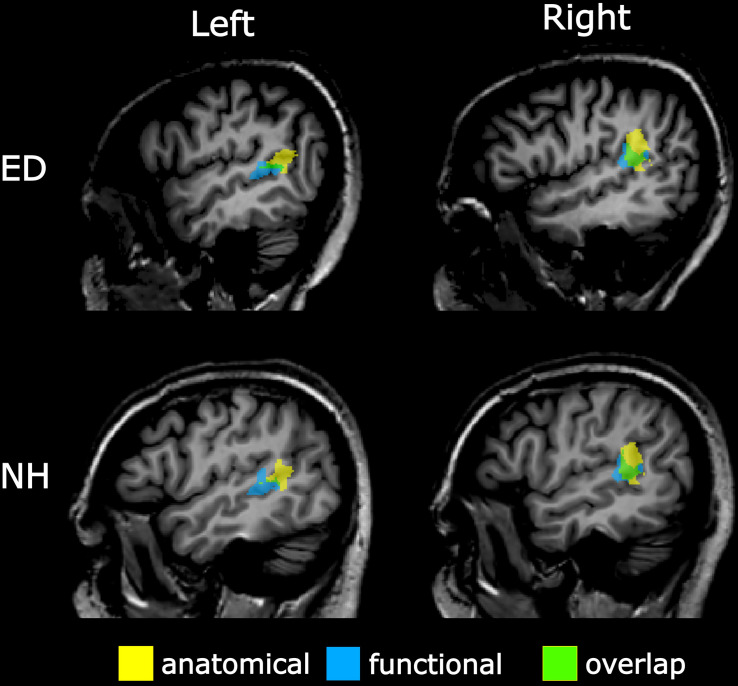
Overlap between anatomically and functionally defined pSTS region. Sagittal view of left (left panel) and right (right panel) pSTS regions defined anatomically via the AICHA (yellow) and functionally via a visual–motion localizer (blue) on a representative ED (top panel) and NH participant (bottom panel). Overlapping voxels between anatomical and functional pSTS regions are displayed in green.

To verify findings from the functionally defined pSTS region, an anatomically defined posterior STS region based on the Atlas of Intrinsic Connectivity of Homotopic Areas (area label 88; Joliot et al., 2015) was also generated and transformed to Talairach space. This atlas-based pSTS ROI also reduced any bias conferred by using a visual motion localizer to functionally define pSTS. The atlas-defined pSTS region was inspected on each individual’s volume, and voxels that extended to the TPJ, parietal operculum, or lateral fissure were removed. Group averaged central voxels and total voxel number are presented in Table 2. The more anterior functionally defined pSTS region had marginal overlap with the more posterior atlas-based pSTS region in both ED (right: 32.91% ± 2.81%, left: 15.36% ± 4.70%) and NH (right: 33.36% ± 4.16%, left: 14.89% ± 4.80%) as can be seen in Figure 3.

### General Linear Model Analysis

To quantify differences in the blood oxygenation dependent level (BOLD) response to tactile motion between ED and NH participants, responses to tactile motion vs. baseline were computed for each participant within each ROI. Significant responses were quantified using a threshold of qFDR < 0.05. The proportion of voxels within each ROI with a significant response was reported.

### Population Receptive Field Analysis

Voxels within all ROIs were analyzed using methods originally developed for retinotopic mapping (Dumoulin and Wandell, 2008) and later modified for tonotopic mapping (Thomas et al., 2015). Using custom software written in MATLAB, we adapted the pRF analysis for our current experimental stimulus, tactile motion. Briefly, we assumed each voxel within a specified ROI had a one-dimensional Gaussian sensitivity profile (or pRF) centered on the preferred direction of tactile motion. For each voxel, we generated a predicted time-course by convolving the pRF with a general hemodynamic response function (Talavage and Edmister, 2004) and the stimulus sequence. The correlation was estimated between this predicted pRF time-course and the actual functional magnetic resonance imaging (MRI) time-course for each of the four scans, and the maximum correlation value and parameters associated with it were extracted. These best-fitting parameters were used as the initial parameters for a non-linear search algorithm (MATLAB’s fminsearch function), which uses unconstrained non-linear minimization to estimate the pRF parameters [center and standard deviation (SD)] that maximize the correlation between the pRF predicted fMRI time-course and the observed BOLD time-course. This procedure was performed for each voxel within the ROI, and the parameters (center and SD) associated with the best-fitting pRF were extracted. The center and SD of the pRF provide estimates for the preferred direction and size of the receptive field for the voxel, respectively. Each direction of tactile motion was assigned a numeric label in a clockwise manner in order to perform the analysis: rightward motion = π/2; downward motion = π; leftward motion = 3π/2; upward motion = 2π.

To be retained for further analysis, a voxel had to meet all of the following criteria, similar to those used in Thomas et al. (2015) and Huber et al. (2019a, b): (1) The correlation between the observed fMRI-time-course and the predicted time-course was greater than *r* = 0.16; (2) the center of the best-fitting pRF fell within the range of tested directions (π/2 - 2π); and (3) the SD of the best-fitting pRF fell within a range based on the interval of our numerically labeled directions (π/2 - 3π/2).

Note that during scanning sessions, tactile motion was simulated by placing the JVP dome on the middle of the index finger pad and moving it in the appropriate direction (Figure 1). For the up direction, the dome was moved toward the body away from the fingertip, whereas for the down direction, the dome was moved away from the body toward the fingertip. There is a substantial concentration of tactile receptors at the fingertip compared to the more proximal region of the finger pad while the amount of tactile receptors on the lateral and medial areas of the finger pad is much more equivalent (Johansson and Vallbo, 1979). As described in the “Results,” the drastically limited number of voxels displaying sensitivity for the up direction led us to exclude it in subsequent analysis on tuning widths.

### Statistical Analysis

Within each ROI, we performed a leave-one-out cross-validation procedure to assess the reliability of our pRF model. For this analysis, we trained the pRF model using all but one scan and found the correlation between the predicted and the obtained time-courses for each left-out scan. Voxels with an average correlation of *r* > 0.16 were retained for subsequent analysis and classified as “directionally tuned.” These procedures were performed separately within each ROI for each participant.

To determine group differences between the proportions of voxels that showed significant activation during tactile motion and for voxels that demonstrated directional tuning, the non-parametric, Wilcoxon rank sum test was used because the data violated normality assumptions. A Wilcoxon rank sum test was also used to compare activation differences between left and right somatosensory ROIs within each group. However, as parametric assumptions were met for tuning width data, mixed analyses of variance (ANOVAs) were used to evaluate the effect of direction and of group in tuning width estimates. Statistical tests were Bonferroni corrected for multiple comparisons when appropriate. Statistical analysis was performed in R version 1.1.463. In addition, because of limited sample size of *n* = 7 per group, *post hoc* power analyses were performed in G^∗^Power 3.1 software. For our statistically significant findings of increased activation for tactile motion in functionally defined right STS and broader tuning widths in functional and anatomical right STS, *post hoc* power was ≥95.71%.

## Results

**FIGURE 4:**
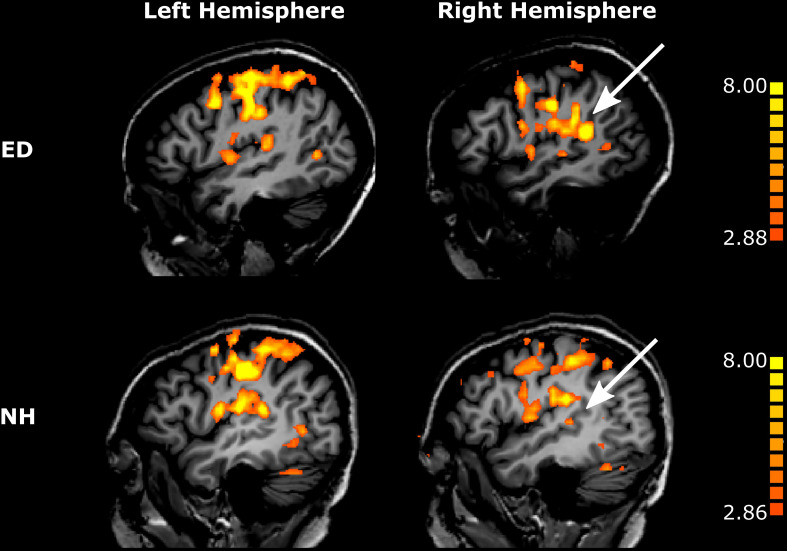
Tactile motion activates right pSTS region to a greater extent in ED than in NH. Voxels with significant activation (qFDR < 0.05) are shown on sagittal sections in the left hemisphere (left column) and in the right hemisphere (right column) for a representative ED (top row) and NH participant (bottom row). White arrows point to right pSTS region.

**FIGURE 5:**
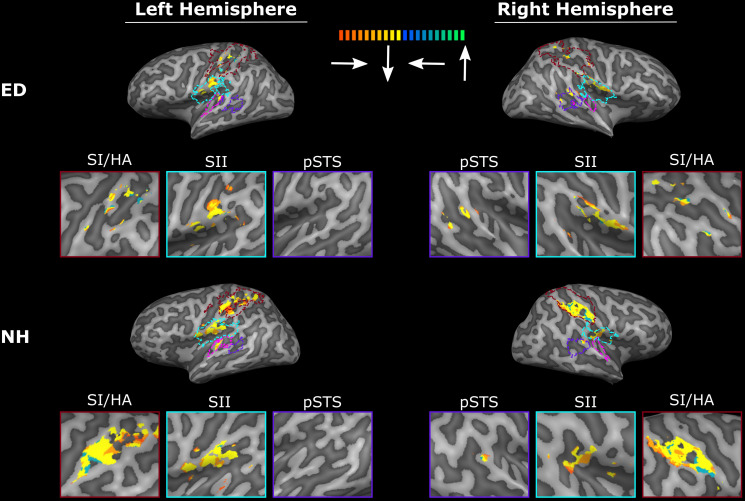
ED and NH participants exhibit similar directional tuning in ipsilateral SI/HA and bilateral SII with minimal tuning in right pSTS region. The best directions within SI/HA (red), SII (blue), functionally defined pSTS region (purple), and PAC (pink) are shown on the cortical surface of a representative ED (top panel) and NH (bottom panel) participant. Direction center values are color-coded along a gradient with right corresponding to red-orange, down corresponding to yellow, left corresponding to blue, and up corresponding to green. All maps are thresholded at *r* > 0.16.

First, we sought to identify regions that were recruited during simulated tactile motion of the right index finger in our ED and NH groups using GLM. To quantify the extent of activation, we calculated the percentage of voxels that showed significant BOLD activity (qFDR < 0.05) within our functionally defined pSTS region and anatomically defined (SI/HA, SII, PAC, pSTS) ROIs for tactile motion simulation vs. baseline periods. Results from this GLM analysis are displayed in Table 3 and Figure 4 and are subsequently referred to as tactile motion responses. We then characterized the directional tuning of voxels within each ROI using a modified pRF model based on the four directions of tactile motion used in this study. Results from our pRF analysis are displayed in Table 4 and Figure 5 and are subsequently referred to as directionally tuned responses.

**TABLE 3:** Percentage of voxels with significant tactile motion response in ROIs anatomically defined (SI/HA, SII, PAC, and pSTS) and functionally defined pSTS region.

ROI name	Hemisphere	NH	ED
SI/HA	R	29.47% (3.47%)	22.63% (4.84%)
	L	44.39% (5.69%)	35.64% (4.74%)
SII	R	23.76% (3.46%)	27.94% (5.67%)
	L	39.56% (7.03%)	41.57% (5.04%)
PAC	R	7.09% (2.97%)	10.80% (4.35%)
	L	13.07% (4.10%)	18.61% (5.62%)
pSTS region (functional)	R	11.96% (2.52%)	47.68% (7.80%)
	L	10.95% (5.30%)	29.34% (9.08%)
pSTS region (anatomical)	R	9.74% (3.22%)	30.28% (5.35%)
	L	15.10% (6.34%)	17.96% (4.25%)

*Numbers represent the group average percentage of voxels within the respective ROI that showed significant activity (qFDR < 0.05) to the tactile motion stimulus vs. baseline. Numbers in parentheses are standard error. Rows represent ROIs.*

**TABLE 4:** Percentage of voxels that demonstrated significant directional tuning within ROIs anatomically defined (SI/HA, SII, PAC, and pSTS) and functionally defined pSTS region.

ROI name	Hemisphere	NH	ED
SI/HA	R	9.07% (3.03%)	2.84% (0.52%)
	L	15.10% (3.28%)	3.79% (1.10%)
SII	R	7.22% (2.86%)	4.42% (2.04%)
	L	12.18% (3.35%)	5.15% (1.69%)
PAC	R	1.30% (0.67%)	0.99% (0.29%)
	L	2.47% (1.88%)	1.80% (1.06%)
pSTS region (functional)	R	3.93% (1.71%)	10.28% (2.68%)
	L	2.10% (1.20%)	3.31% (2.45%)
pSTS region (anatomical)	R	1.76% (0.52%)	3.96% (1.13%)
	L	2.56% (1.18%)	1.38% (0.54%)

*Numbers represent the group average percentage of voxels within the respective ROI that passed our predefined criteria following pRF estimation. Numbers in parenthesis are standard error. Rows represent ROIs.*

### Similar Tactile Motion Response in SI/HA and SII With Reduced Directional Tuning in Contralateral SI/HA in Early Deaf Individuals

As expected, there was no significant difference between ED and NH in the proportion of voxels displaying significant tactile motion response in right SI/HA (Wilcoxon rank sum test, *p* = 0.209) and in left SI/HA (Wilcoxon rank sum test, *p* = 0.456). This finding was consistent for right SII (Wilcoxon rank sum test, *p* = 0.710) and left SII (Wilcoxon rank sum test, *p* = 0.456). As seen in Figure 4, there was a trend toward greater activation in the left somatosensory areas compared to right for both ED (right: 22.6% ± 4.84%; left: 35.6% ± 4.74%) and NH (right: 29.5% ± 3.47%; left: 44.39% ± 5.69%), likely due to tactile stimulation of the right index finger, however, this was not significant for either group in SI/HA (Wilcoxon rank sum tests, *p*’s ≥ 0.07) or in SII (Wilcoxon rank sum tests, *p*’s ≥ 0.07).

Next, we sought to examine the directionally tuned response of voxels within these somatosensory areas that are known to exhibit direction selectivity to process tactile motion (Pruett et al., 2000; Pei et al., 2011). While no particular organization for direction specific voxels is evident in either the SI/HA or SII ROIs shown on the surface maps of ED or NH (red and blue outlines on surface and corresponding red and blue boxes in Figure 5), the sensitivity of this method is apparent as voxels demonstrating directional tuning to three of the tested directions (right, left, and down) are observed. The absence of directional tuning for the upward direction (and exclusion for analysis) is likely due to the procedural drawback discussed in “Materials and Methods.” Briefly, there was reduction of tactile receptors at the proximal part of the fingertip stimulated during the up direction as compared to the distal, medial, and lateral areas of the finger pad stimulated during the down, right, and left directions, respectively (Johansson and Vallbo, 1979). As expected, there was no significant difference between ED and NH in the proportion of voxels within right SI/HA (right: Wilcoxon rank sum test, *p* = 0.179) or SII (right: Wilcoxon rank sum test, *p* = 0.318; left: Wilcoxon rank sum test, *p* = 0.128) that exhibited directional tuning. However, NH had a significantly larger proportion of directionally tuned voxels in left SI/HA as compared to ED (Wilcoxon rank sum test, *p* = 0.006), which survived Bonferroni correction (*p* = 0.00625; 0.05/8).

**FIGURE 6:**
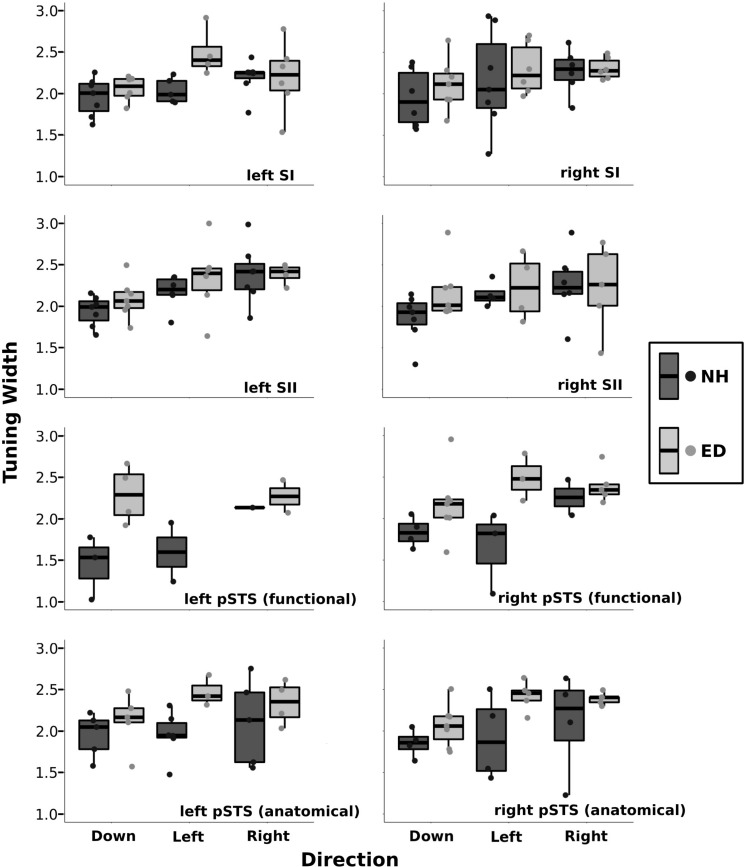
Tuning width estimates are shown separately for down, left, and right directions within the primary and secondary somatosensory ROIs as well as for the functionally and anatomically defined pSTS regions. Boxplots are displayed for each group (NH: dark gray, ED: light gray) where the lower and upper hinges correspond to the 25th and 75th percentiles, and the central bar corresponds to the median. The upper and lower whiskers extend to the largest and smallest, respectively, value no further than 1.5 × the interquartile range. Individual data are also plotted for NH (dark gray circles) and ED (light gray circles).

Figure 6 presents boxplots along with individual data points of the tuning width estimates from the right, left, and down directions for ED and NH groups within various ROIs. A repeated-measures ANOVA was performed with group (ED vs. NH) as a between-participants factor and direction (right vs. left vs. down) as a within-participants factor. It should be noted that not all participants had voxels tuned for all three directions. There was no difference in tuning width estimates between ED and NH groups for the right SI [*F*_(1, 32)_ = 0.77, *p* = 0.386], left SI *F*_(1, 29)_ = 3.15, *p* = 0.087], right SII [*F*_(1, 27)_ = 1.08, *p* = 0.307], or left SII [*F*_(1, 31)_ = 0.734, *p* = 0.398] ROIs. Further, there was no effect of direction on tuning width estimates for right SI [*F*_(2, 32)_ = 1.67, *p* = 0.204], left SI [*F*_(2, 29)_ = 2,79, *p* = 0.077], or right SII [*F*_(2, 27)_ = 1.3, *p* = 0.289]. However, there was a difference in tuning widths based on direction in left SII [*F*_(1, 31)_ = 5.93, *p* < 0.008] that survived the Bonferroni-corrected *p-*value of 0.0083 (0.05/6). Follow-up paired *t*-tests using Bonferroni-adjusted *p-*values demonstrate that tuning width estimates for the down direction were narrower than both the right and left directions (*p*’s < 0.018).

### Early Deafness Resulted in Greater Recruitment of Right Posterior STS Region for Tactile Motion Processing

While recruitment of the left pSTS region in ED as compared to NH during tactile motion stimulation was not significant (Wilcoxon rank sum test, *p* = 0.318), the right pSTS region exhibited significantly greater tactile motion activity in ED compared to NH using a Bonferroni-corrected *p-*value of 0.00625 (0.05/8) (Figure 4) (Wilcoxon rank sum test, *p* = 0.001). This finding cannot be attributed to the size of the right pSTS region as there was no significant difference between groups (Wilcoxon rank sum text, *p* = 0.122). However, as the pSTS ROI was functionally defined with a visual motion localizer and thus may bias toward the ED group (see “Materials and Methods”), we additionally created an atlas-based pSTS ROI (Joliot et al., 2015) to provide a secondary method of comparing tactile motion responses between ED and NH groups. In line with findings from our functionally defined pSTS region, there was no difference between groups in the anatomically defined left pSTS region (Wilcoxon rank sum test, *p* = 0.522). While ED continued to demonstrate greater tactile motion responses in the anatomically defined right pSTS region (Wilcoxon rank sum test, *p* = 0.017), this did not survive multiple-comparisons correction.

A significant number of voxels with direction-selective responses was also observed in functionally defined right pSTS region of the ED group (*mean* = 10.28%, *SE* = 2.68%), but not in the NH group (*mean* = 3.93%, *SE* = 1.71%) as observed in Figure 5 (purple outline on surface and corresponding purple box). A repeated-measures ANOVA revealed that ED participants had significantly broader directional-tuning bandwidths than NH in functionally defined [*F*_(1, 18)_ = 9.91, *p* = 0.0056] right pSTS region. This was further supported by a secondary analysis also showing a significant effect of group in the anatomically defined right pSTS region [*F*_(1, 23)_ = 6.00, *p* = 0.022]. However, given the individual variability within the groups, the present findings on comparisons of directional tuning properties within right pSTS region should be interpreted with caution. This is particularly evident in Figure 6, which shows a reduced number of tuning width measures within pSTS region ROIs compared to somatosensory ROIs as not every participant exhibited voxels with a significant directionally tuned response for all three directions in pSTS.

A closer look at the ED group reveals that four participants had between 14.62 and 16.95%, whereas three had a more limited proportion of voxels with directional tuning (≤3.79%). In the NH group, one participant showed 13.04% of functionally defined right pSTS region voxels with a directional-selective response, whereas the remaining participants had ≤ 5.60% (two participants had 0%) of functionally defined right pSTS region voxels with significant directional tuning. Interestingly, these same four ED and one NH participants showed the largest proportions of direction-selective voxels within the anatomically defined right pSTS regions, albeit smaller values (4.44–9.14, and 3.17%, respectively), whereas the remainder of ED and NH subjects had minimal proportions of directionally tuned voxels (<2.27% and < 2.84%, respectively). In functionally defined left pSTS region, few participants showed voxels that were tuned to the left (NH: 2, ED: 0), right (NH: 1, ED: 2), and down (NH: 3, ED: 4) directions (Figure 6); therefore, no statistical test was conducted comparing groups or tuning widths based on direction in the left hemisphere ROI.

### PAC Is Minimally Involved in Tactile Motion Processing as a Consequence of Early Deafness

As is evident in Figure 4, PAC showed minimal tactile motion response in both NH (right: *mean* = 7.09%, *SE* = 2.97%; left: *mean* = 13.07%, *SE* = 4.10%) and ED (right: *mean* = 10.80%, *SE* = 4.35%; left: 18.61%, *SE* = 5.62%). While group averages trended toward greater activation in PAC for the ED over NH participants (Table 3), this did not reach significance for either hemisphere (right: Wilcoxon rank sum test, *p* = 0.653; left: Wilcoxon rank sum test, *p* = 0.535). In fact, those ED participants who exhibited extant left PAC tactile motion response (4 of 7; > 13%) did not actually reveal activity on Heschl’s gyrus, but instead, this activation was closer to the PT region (Figure 4). Because of the minimal proportion of voxels showing directionally tuned responses within right and left PAC for both groups (≤2.47%) (see pink outline on surfaces in Figure 5), we are hesitant to conclude neuronal populations within PAC that display direction-selective responses to tactile motion.

## Discussion

The main aim of the current study was to examine the effects of early auditory deprivation on tactile motion processing. Using a standard GLM approach, we quantified tactile motion response in SI/HA, SII, PAC, and pSTS region. In addition, we employed a modified pRF model to assess tactile motion direction tuning in these areas.

### Similar Tactile–Motion Response in SI/HA and SII With Reduced Directionally Tuned Response in Contralateral SI/HA of Early Deaf Individuals

We found no significant difference between ED and NH groups in the proportion of voxels within SI/HA and SII demonstrating significant tactile motion responses. Research on tactile perception typically focuses on how we are able to integrate various cues of an object we are directly interacting with, such as position, orientation, and shape. SI is the first cortical region in this process, while SII typically performs higher-order functions on the cutaneous information (Hsiao and Gomez-Ramirez, 2011). In primates, it has been demonstrated that both regions contain neurons that have various tuning properties for object features such as curvature (Yau et al., 2013), orientation (Thakur et al., 2006), and direction of motion (Pei et al., 2010, 2011). Further, directional modulation of cortical activity within somatosensory areas has been shown in humans (Wacker et al., 2011). There was no significant difference in the proportion of voxels that demonstrated directional tuning between ED and NH in right SI/HA or in bilateral SII. However, NH individuals did exhibit significantly increased proportions of directionally tuned voxels in left SI/HA as compared to ED.

This finding is consistent with previous findings showing reduced activation or feature specificity in non-deprived regions. In normally sighted but not early blind (EB) individuals, direction of auditory motion could be successfully classified by PT, a region normally dedicated to decoding auditory motion information (Baumgart et al., 1999; Warren et al., 2002; Alink et al., 2012), suggesting loss of auditory directional tuning within PT due to early visual deprivation (Jiang et al., 2014). In addition, there was decreased activation of PAC during combined vocal and non-vocal stimulation vs. silence in EB compared to sighted controls (Gougoux et al., 2009). The reduced directional tuning of ED contralateral SI/HA reported in the present study implies similar intramodal plasticity and may suggest an extended network responsible for general and more efficient sensory processing of intact modalities (Gougoux et al., 2009; Stevens and Weaver, 2009). Alternatively, this may reflect additional areas that partially take over the functional role of SI/HA, similar to hMT + in EB individuals wherein classification of auditory motion direction is possible in EB participants (Jiang et al., 2014).

Our pRF analysis also provided estimates of tuning width for individual voxels. Across both NH and ED participants, neural tuning for the downward direction of tactile motion was narrower than for right and left directions in contralateral SII. Accurate representations of haptic objects are driven by complex activity patterns and interactions within SI and SII initiated by stimulus-specific activations. SII not only receives tactile information in a feed-forward manner from SI, but also via direct tactile inputs from the thalamus, which are thought to even exceed thalamic inputs to SI (Mackie et al., 1996; Rowe et al., 1996; Tommerdahl et al., 2010). These various inputs likely contribute to the narrow and specific tuning for specific stimulus features, such as motion direction and spatial orientation, underlying the higher-order tactile processing roles of SII neurons (Hsiao et al., 2002). In addition, the increased density of receptors found at the tip of the index finger compared to the medial and lateral areas of the finger pad (Johansson and Vallbo, 1979) likely contributes to the increased sensitivity for the downward direction reported in the present study. This corresponds to prior studies reporting greater directional acuity for proximal/distal vs. lateral/medial motion (Keyson and Houtsma, 1995) and activity modulation in SI and SII for downward vs. upward diagonal direction (Wacker et al., 2011). However, the precise relationship between peripheral sensor distribution and central tuning properties are beyond the scope of this article.

The tuning width of neural populations has also been associated with perceptual abilities. For instance, narrow directional tuning widths of neurons in macaque MT correspond with more precise perceptual discrimination (Purushothaman and Bradley, 2005), and sensitivity of somatosensory neurons in macaque SI and SII is associated with the ability to discriminate the direction of tactile motion (Pei et al., 2010, 2011). There are contradictory findings regarding any behavioral advantages for ED individuals for processing somatosensory information. Some studies report enhanced tactile abilities in deaf, for example, in a suprathreshold change detection task (Levänen and Hamdorf, 2001), but reduced sensitivity in a temporal discrimination task (Papagno et al., 2016). However, other findings report no difference in tactile frequency discrimination (Levänen and Hamdorf, 2001), tactile detection thresholds (Moallem et al., 2010; Heimler and Pavani, 2014), tactile spatial discrimination (Papagno et al., 2016), and tactile motion discrimination (unpublished data). While we found no difference in the tuning widths of voxels within SI or SII between ED and NH groups, future studies are needed to resolve the disparate findings regarding alterations in haptic perception associated with early deafness.

### Greater Tactile Motion Response in Right pSTS Region of ED Participants

While there was no difference in the extent of SI/HA and SII recruitment, there was a significant increase in the tactile motion response of functionally defined right pSTS region for our ED group compared to NH. This finding was confirmed using the anatomically defined right pSTS (although this only trended toward significance after Bonferroni correction), suggesting that multisensory areas serve as prime targets for compensatory plasticity. Future studies that define subregions of the STS using anatomical landmarks or using a vibrotactile localizer to functionally define pSTS region would provide additional evidence on the functional role of this cortical area. For instance, Venezia et al. (2017) demonstrated a posterior–anterior map along the STS dedicated for processing distinct aspects of visual, audio, and audiovisual speech. Functional and modality sensitivities of STS subareas in ED could further elucidate the neural substrates involved in cross-modal reorganization for tactile motion processing. Polymodal regions already display neural areas responsive to multiple modalities so that, during sensory deprivation, receptors from intact modalities can compensate for the deprivation by enhancing response strength and expand to involve neurons deprived of their preferred input. Indeed, changes to neural response properties within normally multimodal areas have been shown for sensory deprived cats. The anterior ectosylvian cortex (AEC) of the cat is a multisensory region containing bimodal and unimodal neurons responsive to visual, auditory, and somatosensory cues. Visually deprived cats show an increase in the proportion of neurons within AEC that are responsive to auditory and tactile input (Rauschecker and Korte, 1993). Similar findings have been reported regarding the auditory field of the anterior ectosylvian sulcus in cats. Normally, ∼30% of the entire neuronal population modulate their response upon somatosensory input (Meredith et al., 2006), and another ∼30% alter their response during visual stimulation (Meredith and Allman, 2009), indicating the existence of subthreshold multisensory neurons. When early deafness is induced via cochlear lesions, this area exhibits significant cross-modal plasticity with ∼90% of neurons demonstrating modifications in their modality response profiles, likely due to a release on the sensory specificity of existing neural connections (Meredith and Lomber, 2011).

In humans, the STS region, including the middle and posterior temporal sulci, middle temporal gyrus, and STG, has consistently been identified as displaying multimodal response properties. The pSTS region is necessary in integrating auditory and visual information (Calvert et al., 2001; Noesselt et al., 2007) and also becomes active during vibrotactile (Beauchamp et al., 2008) and tactile motion stimulation (Jiang et al., 2015) in hearing adults. As a result of auditory deprivation, the pSTS region undergoes reorganization (Li et al., 2013) and becomes recruited for visual motion processing (Bavelier et al., 2001; Shiell et al., 2015), visual temporal processing (Bola et al., 2017), and tactile frequency processing (Levänen et al., 1998). To our knowledge, this article is the first to report similar compensatory plasticity in right pSTS region for tactile motion processing in ED. Further, the unilateral response to tactile motion of the pSTS region reported here is consistent with prior studies examining this area for visual processing under early deafness. A similar hemispheric bias for the right pSTS region over the left has been shown in ED for detecting moving over static visual stimuli (Vachon et al., 2013) and in the anterior STS region in response to faces (Weisberg et al., 2012) in early deafness.

Despite a significantly larger proportion of voxels with tactile motion response in the right pSTS region in the ED group, this region does not demonstrate a considerable directional-selective response for all participants. While we did find a significant proportion of voxels exhibiting directional tuning within the right pSTS region on the group level, this was not representative of the individual data (three ED participants had ≤ 3.79% of directionally tuned voxels in functionally defined right pSTS region). One possibility for this might be due to a more distributed network of neuronal populations in this region rather than neurons dedicated to processing specific aspects of tactile motion. Indeed, ED individuals do show an effect of attention on activation of pSTS while presented with moving visual dot stimuli (Bavelier et al., 2001). A similar effect may occur in the context of tactile motion. While activation of right pSTS region in ED during tactile motion presentation may reflect increased attention and resources for processing a tactile stimulus, the dispersed number of directionally tuned voxels within pSTS reveals that this region is not necessarily involved in processing discrete features of the tactile stimulus. This is consistent with right pSTS region recruitment for directional vs. non-directional visual motion without specificity for a particular direction in ED adults (Retter et al., 2019). Further, for those individuals with directionally tuned voxels, the ED group displayed significantly broader neural tuning for anatomically and functionally defined right and left pSTS regions regardless of motion direction indicating more global processing of the haptic cue vs. specific processing of motion direction by pSTS. This is further supported when comparing the increased proportion of directionally tuned voxels in the functionally defined right STS compared to the anatomically defined right pSTS for all participants. The functionally defined right pSTS was more anterior and specific to areas also responsive to visual motion in ED, supporting the notion that the functionally defined right pSTS highlights a region that is recruited for supramodal motion processing as a consequence of early auditory deprivation.

### Minimal Tactile Motion Response and Directional Tuning in PAC for ED

When presented with visual motion stimuli, a consistent finding is recruitment of auditory cortex in deaf individuals (Finney et al., 2001; Fine et al., 2005). Similar cross-modal plasticity of PAC is revealed during a visual detection task in the peripheral visual field (Scott et al., 2014). These findings fall in line with the enhanced visual processing abilities reported for deaf individuals, specifically greater attention to the visual periphery (Bavelier et al., 2006; Scott et al., 2014) and heightened sensitivity for detecting and discriminating visual motion (Pavani and Bottari, 2012). However, the current results did not find evidence for substantial PAC activation during tactile motion presentation. Further, the minimal activation that was present was not confined to ED participants, and neither group demonstrated directional tuning within PAC.

One major principle guiding cross-modal plasticity is retainment of function (Bavelier and Neville, 2002; Renier et al., 2014), whereby functional reorganization of a sensory-deprived cortical area is guided by computational fitness, or characteristics that will enable the same functional role of the area, also known as functional constancy (Amedi et al., 2007; Saenz et al., 2008; Jiang et al., 2014). The auditory modality is predominant in processing temporal features, and prior studies showing PAC recruitment by ED adults during a visual rhythm task (Bola et al., 2017) and using vibrotactile stimuli (Auer et al., 2007) support the principle of functional constancy. As the current design used spatial features of the tactile stimulus, it is perhaps not surprising that there was no significant recruitment of PAC in the ED group.

Furthermore, the minimal tactile motion response that was seen in PAC was in both groups and was not localized to Heschl’s gryus but close to PT. Numerous findings implicate PT in the dorsal auditory pathway and show PT activation for spatial feature processing such as motion and spatial change (Alink et al., 2012; Isenberg et al., 2012; Jiang et al., 2014). Indeed, in early deafness, PT has been reported to be active in the context of visual motion (Finney et al., 2001), and cortical density of the PT was associated with visual motion detection abilities (Shiell and Zatorre, 2017). Future studies should use an auditory motion/spatial localizer to functionally define the PT and explicitly investigate this region’s response to tactile motion in ED.

## Conclusion

The current findings provide evidence for compensatory plasticity within right pSTS region of ED adults for processing tactile motion. However, it is important to note that this finding did not survive multiple-comparisons correction when the anatomical definition of the right pSTS region (corrected *p* = 0.07) was used, limiting the scope of our results. Future studies that use a vibrotactile localizer, rather than a visual motion localizer as in the current study, to define pSTS region could strengthen evidence for the group difference in activation. In addition, an increase in sample size could reveal a statistical difference for both ROI definitions and would also increase the robustness of present findings. Finally, we acknowledge the potential drawbacks of manual tactile motion simulation by the experimenter including spatiotemporal variability and overall reproducibility. Future replication of the study involving a mechanical device would address these limitations and may reduce the variability found for directional tuning.

Despite the significant increase in tactile motion response of right pSTS region in ED participants, there was no evidence for enhanced directional tuning. The lack of auditory input to the polymodal STS likely drives the increased recruitment of this region, allowing for increased resources allocated to processing tactile motion albeit with reduced tuning to spatial features of the stimulus (i.e., direction). However, this interpretation should be taken with some caution due to the individual variability and the limited directions we simulated manually. Future studies could use a device for automated stimulus presentation, which would allow precise stimulation in a greater number of directions. Another main finding was the reduced directional tuning in contralateral SI/HA of ED despite similar somatosensory area activation relative to NH suggesting that early deafness leads to modified tuning profiles of neuronal populations within intact primary sensory areas. In summary, early deafness leads to cross-modal recruitment of the innately multimodal right pSTS region, despite absence of enhanced directional tuning, and reduced directional sensitivity of intact SI/HA. Taken together these findings suggest that early auditory deprivation results in a more distributed cortical network with a wider response profile for tactile motion processing.

## Data Availability Statement

The de-identified raw data supporting the conclusions of this article will be made available by the authors upon request.

## Ethics Statement

The studies involving human participants were reviewed and approved by the University of Nevada, Reno Institutional Review Board. The patients/participants provided their written informed consent to participate in this study.

## Author Contributions

FJ designed the experiment. AS and FJ collected the data. AS conducted the statistical analysis with assistance on approach and interpretation from CM, EH, and FJ. AS wrote the manuscript. CM, EH, and FJ critically evaluated the manuscript. All authors contributed to the article and approved the submitted version.

## Conflict of Interest

The authors declare that the research was conducted in the absence of any commercial or financial relationships that could be construed as a potential conflict of interest.
